# Working Safely in the Trades as Women: A Qualitative Exploration and Call for Women-Supportive Interventions

**DOI:** 10.3389/fpubh.2021.781572

**Published:** 2022-01-26

**Authors:** Hannah M. Curtis, Hendrika W. Meischke, Nancy J. Simcox, Sarah Laslett, Lily M. Monsey, Marissa Baker, Noah S. Seixas

**Affiliations:** ^1^Department of Environmental and Occupational Health Sciences, University of Washington, Seattle, WA, United States; ^2^Department of Health Services, University of Washington, Seattle, WA, United States; ^3^University of Oregon Labor Education and Research Center, Eugene, OR, United States

**Keywords:** health, occupational safety, construction, women, psychosocial hazards

## Abstract

**Background:**

Construction work offers women economic advancement and self-fulfillment opportunities, but multiple barriers prevent their increased representation in the industry. This study used qualitative methods to identity key physical and psychosocial safety hazards affecting tradeswomen.

**Methods:**

Three focus groups were held in 2015 with 19 tradeswomen in Washington State. Groups discussed workplace hazards and solutions to make the trades safer for women. Discussions were recorded, transcribed, and two independent reviewers analyzed themes.

**Results:**

Participants identified myriad physical and psychosocial hazards including a dangerous work environment, inadequate personal protective equipment, gender discrimination, and fear of layoff for reporting concerns. Participants identified mentorship as a potential intervention to overcome some of these barriers.

**Conclusion:**

Findings suggest that the industry's work environment can be hostile and unsupportive for women, contributing to tradeswomen's injury risk and psychological distress. Future research and interventions should focus on understanding the relationships between and mediating the negative impact of women's physical and psychosocial workplace hazards. Results from these focus groups inspired a randomized control trial to study the impact mentorship has on decreasing physical and psychosocial hazards for women in construction, and improving retention.

## Introduction

“*There's that feeling of pride when you drive by a building that you know you were a part of, and you kind of feel like you left a part of yourself all over the county.”* For women, the benefits of learning a skilled trade extend from psychological to financial. Non-traditional occupations offer women the opportunity to earn higher salaries than many traditional women-dominated professions. Construction workers in the United States (U.S.) earn an average hourly wage of $25.28—more than twice the median hourly earnings for childcare workers and home health aides (1). Unfortunately, women represent just 3% of all skilled building trade jobs in the U.S., a percentage that has barely changed in almost 40 years, despite hiring goals and anti-discrimination laws (2, 3). Growing concern over a future shortage of skilled workers exacerbates the need to increase women's participation in construction by eliminating entry and retention barriers. For union workers, apprenticeship is often the primary training program for trades work, and historically, these programs lacked outreach to women and men of color (4). Once in apprenticeship programs, women experience higher attrition rates compared to men, citing issues related to lack of familiarity with tools, isolation, and discrimination (2, 5, 6). For certain trades—including electricians, laborers, and plumbers/pipefitters/steamfitters—close to half of women canceled their apprenticeship agreements. For women carpenters the cancellation rate was 70% (5).

Previous studies have documented barriers women face when entering and succeeding in construction trades, noting that a dangerous physical workplace and hostile culture contribute to their low retention numbers. Women are between two and five times more likely to sustain upper body sprains or strains from their work than men (7). In general, all workers risk being struck by heavy machinery and traffic vehicles, experiencing slips, trips and falls, electrocution, musculoskeletal disorders, and exposure to toxic chemicals (8–10). Added risk may come from “macho” social norms, which can pressure workers to disregard safety procedures and push their bodies in ergonomically unsound ways (11, 12). Navigating all of these challenges requires focused concentration and vigilance, which can be compromised by psychological stressors (13).

Workplace harassment, itself influenced by organizational and structural factors, can lead to adverse psychological and physiological outcomes for women (14). In addition to sexual harassment, prior studies reveal that inadequate and unavailable personal protective equipment (15, 16); lack of hands-on training with tools of the trades (11); physical overcompensation due to the need to prove their worth (11); lack of available and sanitary restroom facilities; and gender discrimination (4, 12, 17) are among the major safety concerns for tradeswomen. Goldenhar et al. (18) showed an indirect relationship between psychosocial stressors such as discrimination and harassment, and injury or psychological strain, although no gender differences were found. Curtis et al. (19) report the significant impact of psychosocial stressors on injury risk for women: 31% of tradeswomen reported high perceived stress compared to 18% of men, and nearly double the rate of injury at work in the past year. Turner et al. (20) highlight access to support networks as a form of resilience for working tradeswomen: however, lack of formal and informal structures, as well as from male coworkers, to facilitate such support in the workplace are lacking. While these findings highlight the ongoing need to support tradeswomen's health and safety needs, as well as further research to better understand their root causes, the present study provides needed qualitative perspectives from current tradeswomen that provide a roadmap for meaningful intervention to increase workplace safety and boost representation of women in the trades.

The goal of this work was to: (1) explore specific physical and psychosocial workplace hazards for tradeswomen, and (2) shed light on the impact of physical and psychosocial workplace hazards on tradeswomen's health and safety to inform intervention research needed for improving workplace safety and health.

## Materials and Methods

We employed a collaborative, community-based participatory approach to our research by partnering with non-profit group **[organization n name REMOVED for peer review]**. An advisory committee with members of the partner agencies met with the investigator team monthly in early 2015 to discuss the project's scope, develop guiding questions for focus group discussions, and assist with recruitment. The advisory board included a labor educator, members of **[organization name REMOVED for peer review]**, as well as **[institution name REMOVED for peer review]** faculty and research staff.

### Sample Selection

Three focus groups were held in March and April 2015, with a total of 19 tradeswomen. Each group was held in a different region of Washington State: Seattle, Vancouver, and Spokane, to increase generalizability of results.

Given the very low numbers of women in the trades, a purposive sampling strategy was used to recruit focus group participants using contacts recommended by community advisory committee members and union partners. Committee members provided the research team with a list of tradeswomen who were then contacted via phone and email. Eligible participants were currently working or had worked in one of the building and construction trades in Washington State. Recruitment letters included an overview of the study's purpose, group logistics, and assurance of the study's voluntary and anonymous nature. Twenty-seven women agreed to participate in a focus group; however, eight women were absent due to illness or unexplained reasons. Considering tradeswomen's demanding schedules, hearing from 19 participants was noteworthy.

### Data Collection

The study advisory committee developed the focus group questions using information from previous research as well as concerns raised by partner organization **[organization name REMOVED for peer review]**. Signed informed consent was obtained from each subject prior to participation. All data were collected anonymously and groups were audio recorded to allow for verbatim transcription. Two research staff affiliated with the study attended and took additional notes. Each group lasted ~2 h with a short break and was held at convenient times for the workers. Food was provided and participants received $50 as compensation for their time. The **[institution name REMOVED for peer review]** declared the project exempt from review.

The moderator, a labor educator and advisory committee member with experience in group facilitation, facilitated all three focus groups. She opened by asking participants to discuss the positive aspects of their jobs before focusing on physical and psychosocial risks that women experience at work (see Table 1).

**Table 1:** Focus Group Facilitation Guide.

**Primary question**	**Possible probes**
1. What is the best thing about working in the trades?	
2. What are physical risks you experience at work?	a. Do you experience dust? Loud noise? Heat? Toxic chemicals? Machinery? Musculoskeletal disorders?
3. What are risks that threaten your wellbeing at work?	a. What do you find stressful at work?
4. Which risks affect you specifically as women?	a. Do you experience sexual harassment? Discrimination based on gender, race, or sexual orientation? Isolation? b. What about balancing work with your home and childcare responsibilities? c. Do you feel like you have to prove yourself?
5. What are some ways to reduce these problems?	a. What are some things you yourselves can do? b. How can your coworkers help? You supervisor? c. What more would you like to see done?
6. Of all the risks we talked about today, which ones do you think are the most important to address?	

### Data Analysis

Audio-taped data from each focus group were transcribed along with observer notes. A conventional approach to content analysis was used to derive codes from the transcripts and written sheets (20). HC developed a codebook, and HM and HC independently read the transcripts line by line to identify codes inductively. Codes were then synthesized to summarize key themes to identify larger concepts and were categorized into themes. HM and HC discussed their discrepancies until consensus was reached, which increased the validity of the findings. Subsequently, the data were further validated by having the study's advisory committee review the themes. These procedures are similar to qualitative methods used in other studies (21, 22). Following initial analysis, the themes were quantified based on frequency of appearance during the discussions. We report on themes that appeared in all groups five or more times.

## Results

### Study Participants

Eight women participated in the Seattle focus group, six in Spokane, and five in Vancouver. Participants represented a variety of trades and career levels: carpenters (*n* = 4); drywall finisher (*n* = 1); electricians (*n* = 8), laborers (*n* = 4), operating engineer (*n* = 1) and plasterer (*n* = 1). Three participants were apprentices, two were retired tradeswomen and 14 were journey-level tradeswomen (i.e., experienced workers who completed an apprenticeship program and continue to work), several of whom were in supervisory positions. To protect participant confidentiality, we did not collect demographic data beyond trade, and career level; however, group discussions revealed that all but one participant were union-affiliated workers. Although the study team did not ask participant's age, the potential range among focus group participants (from age 18 to 65 or older) may have impacted the concerns and perceptions of workplace hazards among these informants.

Table 2 lists themes identified most frequently across all three focus groups. Themes were categorized as either threats to tradeswomen's physical health or psychosocial wellbeing. Here, we discuss the themes based on our analysis, using quotes where appropriate.

**Table 2:** Themes about Tradeswomen's Workplace Risks (in order of frequency, most to least).

**Physical threats**	**Psychosocial threats**
Nature of the job: dangerous work environment	Inadequate bathrooms
Physical limitations	Gender discrimination
Inappropriate personal protective equipment	Unequal treatment
Overcompensation	Harassment
	Fear of layoff for reporting concerns
	Work-life balance

## Physical Threats

### Nature of the Job: Dangerous Work Environment

Participants reported acute and chronic injuries sustained during the course of their normal work: injuries from heavy power tools; being hit by tools dropped from scaffolding; and having to work in tight spaces without a hard hat. These occupational hazards were the first issue workers raised when asked about their physical risks. Participants said that strains and sprains from carrying heavy loads were often unavoidable in construction, especially for women who were not trained properly. Exposure to toxic chemicals was another concern, although many participants only became worried during pregnancy. Every electrician in our groups mentioned the dangers of electrocution. While they pointed out that electricity “*doesn't care what gender you are,”* they noted increased risk for women without proper gloves or training.

In addition to acute injuries, participants also discussed chronic injuries from the everyday physical aspects of their work. “*…The normal wear and tear of it—I mean I've only done it for 2 years, and I can already feel it in my back and my knees” (Apprentice electrician)*.

### Physical Limitations

Participants noted a variety of physical limitations they must negotiate while performing required tasks on the job. Women's smaller stature, compared to men, can affect their ability to do the physically demanding work safely. One apprentice described, “*Pipe bending for example…you got a lot of large men who are bending these pipes… And my first day of pipe bending, since I'm vertically challenged…I was flying across the room…because I couldn't get the leverage to bend the pipe.”* While this apprentice laughed about her experience, she highlights an important safety concern for tradeswomen. Their physical stature is especially dangerous when combined with training based on men's ergonomics, a “macho” work culture, and pressure to overcome sexist stereotypes about women being weak. The groups discussed how certain tasks and tools are harder for them due to their size and relatively lower upper body strength compared to men. One electrician shared that her company had to special order a taller ladder for her, which was then more difficult to carry. Several apprentices described trouble they faced learning to use heavy tools designed for men's bodies.

### Inappropriate Personal Protective Equipment

A dominant theme was experiencing injury due to ill-fitting personal protective equipment (PPE). Participants reported that most jobsites do not provide PPE that fit them properly, including safety harnesses, coveralls, boots, and safety glasses. One journey-level laborer called attention to the danger standard size harnesses can pose for tradeswomen: “*The harnesses—safety harness for tying off…they're not made for women. You would have to buy a specific one for female's bodies. They don't fit you right. If you were to fall off a building with a standard harness on, it would do more damage than good.”* Participants also shared stories about jerry-rigging their own harnesses and equipment, including a carpenter apprentice who said she uses her tool belt as a makeshift harness. In addition to potentially not being properly designed or certified, such worker-supplied PPE is not always allowed on jobsites.

The issue of ill-fitting gloves came up frequently, with women noting that they are often unable to perform technical tasks wearing the standard issued gloves. One journey-level electrician exclaimed, “*Oh the gloves! Yeah, they never have gloves small enough.”* Several participants don't wear gloves on the job because the poor fit hinders their ability to work.

In addition to physical risks, participants described psychosocial threats to their health and safety, including psychological stressors. These frequently related to a workplace culture that minimizes women's needs and distrusts their presence on jobsites.

## Psychosocial Threats

### Inadequate Bathrooms

In all groups, participants acknowledged the challenge of urinating outside as many of their male coworkers do. They also expressed dissatisfaction with the cleanliness of jobsite porta-potties and scarcity of women-only bathrooms. A journey-level electrician stated, “*I just don't use the bathroom. I mean, I only work like 5 min from my house so it's like I will hold it all day long. I hate it—I cannot stand Honey Buckets.”* Lack of running water was also a problem for women who are menstruating. As a journey-level laborer said, “*I don't think I've ever been on one job that's actually had a portable sink.”*

Reluctance to voice their concerns due to men's anger over perceived “special treatment,” as well as distaste for unclean facilities leaves many women with few choices: using unsanitary porta-potties that may lack locks or be inaccessible depending on the size of the jobsite; traveling off site to commercial facilities; or not using bathrooms at all during their shifts.

### Gender Discrimination

#### Unequal Treatment

Participants agreed that “earning your voice” is critical for success in the trades but that it's more challenging for women who are often seen as incapable. Disproving sexist cultural norms—that women don't belong in the trades, that they are weak, and that they don't work as hard as men—was paramount to our participants. Unfortunately, some of that sexism becomes internalized. Several participants mentioned not wanting to work with other women because they believe women coworkers won't work as hard as men.

Many journey-level participants believe that they had not received training equal to that of men during their apprenticeships, especially when it came to hands-on opportunities with tools and machinery:

“*The heartbreak about onsite job accidents is someone who's new to the trade that was withheld the training and information from the journey-level workers around them. And while this happens to a lot of new people it specifically and oftentimes uniquely happens to women and minorities in the trades. They are not told all the safety concerns of their trade, or how to do something safely, but left out to fend for themselves because there is a group of people who don't think they should be there” (Retired electrician)*.

Groups also discussed the discrimination women face in securing work hours. Despite making similar hourly wages to men, participants reported earning less overall due to companies and unions preferentially hiring men.

#### Harassment

While the older journeywomen agreed that harassment has decreased over the past thirty years, other participants said that it continues to be a significant issue. Both experienced and newer workers shared stories detailing a range of harassing behaviors, such as men putting up nude photos of women in the break rooms, telling obscene jokes, and physically groping tradeswomen. Some behaviors, including being asked to fetch tools that don't exist, were seen as obnoxious forms of hazing, which all new workers said they experience. The women said they either laughed off the hazing or accepted it as normal. However, for many participants, “friendly hazing” becomes “harassment” when they are targeted for their gender. One journey-level carpenter noted, “*There's [men] that just basically say, “whatever I can do to make your life miserable, and possibly get you off this job, I'm gonna do it.””*

Most of the discussions focused on what participants described as “harassment” rather than “friendly hazing.” How women responded varied based on the level of threat and their relationships with their coworkers. This included ignoring the behavior, laughing along, or fighting back. In the case of the nude women calendar, one participant hung up a calendar with pictures of semi-nude men, which embarrassed her male coworkers enough to remove all nude pictures. Another woman described a situation where the physical environment and workplace hierarchy enabled her harasser:

“*So I ended up with this journeyman who—he was doing this work up in the attic and he would make me crawl in front of him. I had never been in this attic before but I had to crawl in front of him as we're doing this job. The only thing I can guess is cause he wanted to watch me crawl from behind! …” (Journey level electrician)*.

An electrician apprentice shared a story about being forced to work with a journeyman who constantly caused her to fear for her safety. “*Yeah, he was predatory, like scary. So—and I didn't know what to do.”* While her supervisor resolved the issue once the apprentice spoke up, she felt threatened and alone for over a month.

### Fear of Layoff for Reporting Concerns

Despite unfair practices and harassing behavior, women were reluctant to voice their concerns. Many participants had heard stories about tradeswomen who were laid off for speaking up to management about their safety needs. As a journey-level electrician stated, “*And if you ask for that [handwashing station], which you're also entitled to, you're on that layoff next week too.”* These stories perpetuated a real fear that women would be forever “marked” and punished for signaling discriminatory practices by their coworkers or companies. “*Companies don't like complainers”* was a common refrain during the groups. One journey-level laborer described the high stakes of speaking up through other's stories: “*[Filing a sexual harassment complaint is] a stigma. I mean there's a woman from [town] who sued and that's all you ever hear…she never worked again…”*

### Work-Life Balance

Eight participants mentioned having children that they care for in addition to their full-time work in the trades. Balancing unpaid family responsibilities with physically and emotionally demanding trades work created additional stress. Participants lamented the lack of paid sick leave in the industry at the time, which is especially problematic for tradeswomen with children: “*Childcare is tough for working woman. Whether you're a single parent or not, it's the mom that's gotta take care of the kid when they're sick and that's still an issue today for women who miss work” (Journey-level operator)*.

Participants also raised concerns about the nature of construction scheduling, where hours are long and erratic and frequently involve travel.

### Overcompensation

As women, our participants constantly felt the need to prove their worth to coworkers and supervisors who assumed that they were not capable of the physically demanding trades work. Participants told multiple stories of starting at new jobsites and having to earn their crew's respect by being the hardest worker. “*You have to be twice as good,”* was a common refrain. Many participants proudly noted that they were top of their apprenticeship class, which enabled them to reach journey-level status. But being the best in class did not always translate to respect on the jobsite. As several participants stated, “*[Men] pull up and [supervisors] know they can do it. [Supervisors] assume [men] can do it until [men] prove them wrong.”* But for women, the opposite is true. No matter how long they have been in the trades, women are viewed as inexperienced and weak until they prove their worth.

For many tradeswomen, proving their abilities means working twice as hard, often at the expense of their health and safety. One retired electrician shared, “*I also think that a lot of women in that desire to go back and show the boys on the job that they can do it, hurt themselves doing that. They lift too much, they carry too much, they work too hard, they do stuff that takes two people to do to prove that they can do the job, especially to naysayers on the job.”* Because they are fighting against sexist stereotypes, many tradeswomen are reluctant to ask for help when needed, which can increase their risk for injury.

## Discussion

Tradeswomen's workplace experiences appear not to have changed much in the 20 years since Goldenhar et al. (11, 17, 19) reported many of the same physical and psychological stressors. This lack of progress is disheartening but not unique to construction. As evidenced by the recent #MeToo movement and the experiences of discrimination and harassment shared by thousands of women across industries, workplace gender inequity has wide-reaching and devastating implications. Lifting up tradeswomen's voices is an important step in moving toward justice for all workers.

Although all construction workers risk injury, tradeswomen experience unique physical threats and psychosocial stressors that may put them at greater risk for acute and chronic injury, as well as psychological distress. Similar to other studies (3, 11, 23), our focus groups highlight how multiple factors negatively contribute to women's wellbeing at work: exposure to occupational hazards within the jobsite environment; a sexist worksite culture which affects women's willingness to report on unsafe conditions; and pushing their bodies in ways that are injurious to their health in order to perform skilled tasks. These challenges perpetuate a hostile work environment that keeps women's participation low.

Demanding work schedules are a primary cause of women's concern with work-life balance and are part of the broader category of stressors created by their organizations. While the issue of scheduling may appear gender-neutral, it specifically disadvantages women workers who may have more caregiving obligations (24, 25). The “fear of layoff for reporting concerns” theme from our results falls under a larger issue of women lacking agency in the industry. Tradeswomen feel powerless to speak up or to advocate for themselves due to their unstable position and low social capital (3). This apprehension is reinforced by the stories women hear and witness of other women being laid off or stigmatized for voicing concerns about the lack of bathrooms or their coworker's behaviors: examples of how, on a societal level, harassment claims are often ignored and justice for accusers is not served.

### Future Directions for Addressing Tradeswomen's Stressors

Certain stressors, such as the availability of clean bathrooms and better fitting PPE, can be addressed at the policy and regulatory level where federal and state level Occupational Safety and Health Administration regulations might be better enforced. Although, women first need to feel empowered to raise these concerns to management, which necessitates them feeling supported and confident that they will be taken seriously. Other stressors, related to workplace culture and safety climate, could be addressed by interventions targeted at managers and other industry leaders to assist them in creating a safe and supportive work environment, one where harassers are held accountable. Lastly, providing skill-based training for tradeswomen on how to perform tasks safely given their unique physique; on how to communicate safety concerns and take legal steps when such behavior results in unfair treatment; and how to support other women in navigating the pitfalls of working in a male-dominated industry, might ameliorate the negative impact on health as well as retention in the industry.

Findings from the focus groups confirm that efforts to recruit and retain tradeswomen continue to be needed. A direct strategy to meeting this challenge is one that engages with women workers, addressing issues as they arise and creating a supportive environment for them to thrive and advance. Mentorship is already a key component of construction work, as apprentices train under and are supervised by journey-level workers for several years. This relationship is an essential part of apprentices on the job training—how they learn the unwritten rules of the trade—and it affects their experiences with safety. Mentorship has been proven effective in increasing retention in apprenticeship programs for minority populations (4, 5). However, because of the temporary nature of construction work, the relationship between one apprentice and one journeyman may last a short amount of time. Having a mentor who is available past the duration of a single project is important, as is the need for mentorship by someone not connected to the apprentice's jobsite.

Guided by the findings of these focus groups, conversations with key stakeholders, and an ongoing recognition of the need for interventions aimed at improving the experiences of women in the trades, researchers on this manuscript have developed and deployed a mentorship training program to train journey-level workers to mentor female-identifying apprentice-level mentees. An ongoing randomized control trial in collaboration with Sheet Metal, Air, Rail, and Transportation International (SMART) seeks to evaluate the impact this mentorship training program has not only on the physical and psychosocial experiences of women in the trade, but also on their retention in the trade, and their reported development of skills such as self-advocacy, goal setting, problem solving, active listening, and problem solving. Many focus group participants noted that increasing women's representation will go a long way toward making the trades a safer environment. The ongoing mentorship program seeks to improve the health and safety experiences of women in the trades, and decrease dropout of women apprentices, leading to increased representation of women.

## Limitations of the Focus Group Exploration

This study confirmed some known and uncovered some lesser-known health and safety stressors for tradeswomen. Due to the self-selection of a relatively small sample and the exploratory nature of the study it isn't possible to confirm that the themes identified are a comprehensive list of all stressors experienced by *all* tradeswomen, nor that these stressors actually cause poor health. Due to the sampling strategy and challenge of reaching non-union tradeswomen, findings do not represent non-union voices. As we heard from our participants, unions can offer an extra level of security for women entering the trades and support for those advocating for safer working conditions. Additionally, although participants did not disclose their race or ethnicity, the groups appeared to be majority white women. As a result, we are missing the perspective of minority women for whom the dual marginalization of race and gender may be amplifying their experience of both types of harassment (14). The same is true for women who face additional discrimination based on their sexual orientation or gender identity.

Given that many stressors reported in this study have been previously documented, it is reasonable to believe that the same stressors still affect women's experience in the trades, causing many to either leave a decent-paying job or suffer psychological stress and injury risk. Although our study focused on physical and psychological stressors and safety concerns, the women had many positive things to say about their jobs—citing the high wages, skill application, and personal satisfaction in seeing the tangible results of their work—and would not trade them for anything else. As we are currently evaluating with a randomized control trial, efforts should focus on helping connect experienced tradeswomen with apprentices via mentorship, and empowering them with leadership skills to thrive throughout their careers.

## Data Availability Statement

The raw data supporting the conclusions of this article will be made available by the authors, without undue reservation.

## Author Contributions

NS, HC, and HM conceived of and designed the original research study. Data collection tools were developed by HC, HM, and NS. HC and HM undertook data analysis and interpretation. HC and HM drafted the manuscript. HC, HM, NS, LM, and MB undertook critical revision of the manuscript. MB integrated critical information regarding how the focus groups contributed to the design of the ongoing Randomized Control Trial. All authors gave final approval of the version to be published and as such all agree to be held accountable for the work.

## Funding

Funding and support for this project was provided by the Washington State Department of Labor and Industries. SHIP (Safety and Health Investment Projects) is the specific department that funded this study within Labor and Industries.

## Conflict of Interest

The authors declare that the research was conducted in the absence of any commercial or financial relationships that could be construed as a potential conflict of interest.

## Publisher's Note

All claims expressed in this article are solely those of the authors and do not necessarily represent those of their affiliated organizations, or those of the publisher, the editors and the reviewers. Any product that may be evaluated in this article, or claim that may be made by its manufacturer, is not guaranteed or endorsed by the publisher.
